# Digital Pathology and the AI-Based Quantification of the Tumor Microenvironment in Gastrointestinal Cancer: From Tumor Budding and Tumor-Infiltrating Lymphocytes to Tertiary Lymphoid Structures

**DOI:** 10.3390/ijms27104386

**Published:** 2026-05-14

**Authors:** Justyna Łapińska, Klaudia Kasperczuk, Klaudia Kańczugowska, Aleksandra Gałan, Weronika Pająk, Jakub Kleinrok, Ryszard Sitarz, Jacek Baj, Agnieszka Korolczuk

**Affiliations:** 1 Department of Clinical Pathomorphology, Medical University of Lublin, Jaczewskiego 8b, 20-090 Lublin, Poland; lapinskaju@gmail.com (J.Ł.); klaudiakasperczukk@gmail.com (K.K.); kanczugowskak@gmail.com (K.K.); klejs.90@gmail.com (J.K.); 2 Student Scientific Society of Forensic Medicine, Department of Correct, Clinical and Imaging Anatomy, Medical University of Lublin, 20-810 Lublin, Poland; aleksandra.galan00@gmail.com; 3 1st Department of Psychiatry, Psychotherapy and Early Intervention, Medical University of Lublin, Gluska Street 1, 20-439 Lublin, Poland; ryszard.sitarz@umlub.edu.pl; 4 Department of Correct, Clinical and Imaging Anatomy, Medical University of Lublin, Jaczewskiego 4, 20-090 Lublin, Poland; jacek.baj@umlub.edu.pl

**Keywords:** tumor microenvironment, gastrointestinal cancers, tumor-infiltrating lymphocytes, tertiary lymphoid structures, artificial intelligence, digital pathology

## Abstract

Advances in digital pathology and artificial intelligence (AI) are significantly transforming the approach to analyzing the tumor microenvironment (TME) in gastrointestinal cancers (GICs). The TME consists of tumor cells, stromal components, and immune cells. It plays a key role in disease progression, treatment response, and patient prognosis. This review discusses the most important TME biomarkers, such as tumor budding (TB), tumor-infiltrating lymphocytes (TILs), and tertiary lymphoid structures (TLSs), with emphasis on their prognostic and predictive significance. Traditional histopathological assessment of these parameters is limited by subjectivity, intraobserver variability, and time-consuming nature. In this context, AI-based tools enable automated, quantitative, and more reproducible analysis of entire histological sections. Deep learning models allow the accurate detection and classification of structures and also analysis of their spatial organization. They provide new biological insights unavailable in routine diagnostics. The integration of imaging data with molecular and clinical information leads to the development of personalized medicine. Despite numerous advantages, the implementation of AI in clinical practice continues to face challenges related to standardization, data availability, and model interpretability.

## 1. Introduction

Gastrointestinal cancers (GICs) including esophageal, gastric, pancreatic, liver, biliary tract, colon, and rectal tumors represent one of the leading causes of cancer-related mortality with approximately 4.8 million new cases and 3.4 million deaths annually [1,2,3]. Given the significant global challenge of GICs, increasing attention has been directed towards the identification of prognostic biomarkers and ways of early detection.

The tumor microenvironment (TME) contains tumor cells, surrounding cells and secreted factors promoting the influx of non-malignant cells, blood vessels, and stroma. Together, these elements provide a favorable environment for tumor growth, metastatic spread, and response to treatment [4,5,6]. The TME contributes to the avoidance of immune response and resistance to therapy by inhibiting the cytotoxic functions of immune cells [5]. Cells within the tumor, including immune system cells, play a key role in tumor development or inhibition, as well as in tumor response to various treatment methods. As a response to cancer cell apoptosis following radiotherapy or chemotherapy, signaling pathways may be activated. Therefore, the modulation of the interactions in the TME is a strategy for inhibiting tumor growth and completely eliminating clonogenic cells in the TME [7].

Tumor budding (TB) is defined by the presence of isolated single cancer cells or clusters of up to four cancer cells at the invasive tumor front or the tumor center. It is considered to be an emerging prognostic marker in solid cancers, including colorectal cancer (CRC) [8,9]. TB appears to be a morphological reflection of epithelial–mesenchymal transition (EMT), characterized by more invasive and migratory potential linked to therapy resistance and cancer-cell stemness. This altogether makes the presence of TB an unfavorable prognostic factor associated with local recurrence and both lymph node and distance metastasis [9].

Tumor-infiltrating lymphocytes (TILs) define all lymphocytic populations infiltrating the tumor tissue, which are divided into two groups based on their position: Lymphocytes within the tumor and lymphocytes in the tumor stroma, where presence is associated with positive outcomes from immune checkpoint inhibitors (ICIs) [10]. TLS level is an important biomarker for risk stratification and treatment decisions [11].

Tertiary lymphoid structures (TLSs) are described as ectopic lymphocyte aggregates that develop in non-lymphoid tissues in response to chronic inflammation, including those associated with tumors. TLS presence is positively correlated with a reduced risk of recurrence after the surgical resection of solid tumors and is regarded as a predictive indicator of a more favorable response to immunotherapies in melanoma, breast, and lung cancer [6,12,13]. However, the prognostic and predictive role of TLSs in GICs remains inconsistent across studies [13].

In recent years, artificial intelligence (AI) has been successfully used to evaluate described parameters and provide precise pathologic diagnoses [14,15]. Traditional manual assessment of these features is limited by interobserver variability, subjective grading, time consumption and required pathologist training. AI-driven digital pathology offers standardized, high-throughput, and precise quantification eliminating subjectivity and variability in the assessment, analyzing the entire tumor area, providing a more accurate assessment [16].

This review synthesizes recent literature on AI-based digital pathology for quantifying the TME in GICs, with a particular focus on integrating TB, TILs, and TLSs as emerging immunological biomarkers, discussing all crucial TME factors. We have summarized the current application of these indicators and discussed the clinical utility of AI in this field. Moreover, we have discussed the value of the AI-driven quantification of TME features as a transformed modern GIC pathology, as well as its limitations, challenges, and future perspectives. AI-based quantification has been critically discussed with regard to the combination of tumor biology.

Gastrointestinal cancers represent a distinct setting for AI-based TME assessment because they arise in anatomically and biologically diverse organs, including the esophagus, stomach, colorectum, pancreas, liver, and biliary tract [17,18]. This heterogeneity is reflected in organ-specific stromal architecture, inflammatory background, microbiome exposure, microsatellite instability (MSI)/microsatellite stability (MSS)-related immune phenotypes, mucin production, necrosis, and pancreatic desmoplasia [19,20,21,22,23]. Consequently, AI models developed for GICs must address substantial spatial and histological variability, which may limit direct transferability from other tumor types.

The novelty of this review lies in integrating tumor budding, tumor-infiltrating lymphocytes, and tertiary lymphoid structures as interconnected spatial components of the gastrointestinal TME, rather than discussing them as separate biomarkers. This review provides a GIC-centric framework, linking invasive-front morphology, immune organization, AI-based quantification, validation issues, and clinical implementation, distinct from broader reviews on AI in pathology or isolated TME markers [14,24,25,26,27,28,29,30].

## 2. Methodology

This study was conducted as a structured narrative review with thematic synthesis of the literature on AI-based digital pathology for the assessment of the TME in GICs. A comprehensive search for articles published in English between January 2016 and December 2025 was performed using PubMed/MEDLINE, Scopus, and Google Scholar. The search strategy combined keywords related to AI (e.g., “artificial intelligence”, “machine learning”, “deep learning”, “convolutional neural networks”), digital pathology (e.g., “whole slide imaging”, “histopathology”, “computational pathology”), gastrointestinal cancers (e.g., “colorectal cancer”, “gastric cancer”, “pancreatic cancer”, “liver cancer”), and TME-related terms (e.g., “tumor microenvironment”, “tumor-infiltrating lymphocytes”, “stroma”, “tertiary lymphoid structures”).

Titles and abstracts were screened for relevance, followed by the full-text evaluation of eligible articles. Reference lists of selected studies were manually reviewed to identify additional relevant publications. We included original research articles focusing on AI-based analysis of histopathological images in GIC, particularly those addressing key TME components such as TB, TILs, and TLS, as well as studies evaluating spatial organization and tumor–stroma interactions. Review articles were used to provide background context and support the interpretation of findings. Studies were excluded if they were not related to gastrointestinal malignancies, did not involve histopathological image analysis, focused solely on technical model development without biomedical or clinical relevance, or were non-full-text publications (e.g., conference abstracts).

For the included studies, data were extracted regarding cancer type, TME components analyzed, data modality (e.g., H&E, IHC, whole slide imaging), AI methodology (including model architecture and training strategy), validation approach, and reported clinical or biological outcomes. The findings were synthesized qualitatively, with particular emphasis on methodological approaches, spatial analysis of the TME, clinical relevance, and the limitations of AI-based assessment, including issues related to data heterogeneity, lack of standardization, and translational applicability in GICs.

As this was not designed as a systematic review, no formal risk-of-bias scoring or meta-analysis was performed.

## 3. The Tumor Microenvironment in Gastrointestinal Cancers

### 3.1. Biological Concept and Composition of the TME in GICs

The TME in GICs contains tumor cells, surrounding cells including TILs, tumor-associated neutrophils (TANs), tumor-associated macrophages (TAMs), cancer-associated fibroblasts (CAFs), endothelial cells, extracellular matrix proteins, associated inflammatory pathways, a variety of growth factors, chemokines, proteolytic enzymes, and specific biochemical characteristics, such as hypoxia and low pH. Collectively, these components provide an environment for the maintenance of cancer stem cells (CSCs), the spread of cancer cells to metastatic sites, angiogenesis, and apoptosis, as well as the growth, proliferation, invasion, and drug resistance of cancer cells [4,17,31,32].

The components of the TME form a specific spatial arrangement in which different cell types are located in specific regions of the tumor. The tumor core, invasive front, and stroma tissue differ in their cellular composition and biological function. The invasive front is characterized by the highest number of immune cells, including CAFs, which are responsible for the production of growth factors, chemokines, an increased extracellular matrix (ECM), remodeling, and the recruitment of immune cells [33,34]. In contrast, the tumor core contains significantly fewer immune cells, which may indicate local immunosuppression mechanisms and limited antitumor immune response activity. Spatial arrangement is an important characteristic of tumor biology, as TME analysis results may depend on the biopsy site [34].

The spatial distribution of immune cells within the TME is crucial for tumor immunophenotypes and allows for morphological classification into immune-inflamed, immune-excluded, and immune-desert tumors. Immune-inflamed tumors, also referred to as “hot” or “immune-infiltrated”, are characterized by an abundant infiltration of CD8^+^ T cells within the tumor parenchyma, which involves tumoricidal immune attacks. The immune-inflamed phenotype, accounting for up to 50% of all human tumors, is generally associated with favorable chemotherapy and IBS response, elevated genomic instability, and antigenicity. In immune-excluded tumors, CD8^+^ cells are trapped in peritumoral stroma, failing to directly eliminate tumor cells, which results in ineffective T cell activation, proliferation, and trafficking. Immune-desert tumors, so-called “cold” or “ignored”, are characterized by CD8^+^ cells being barely present in both parenchymal and stromal sites. This immunophenotype presents an unfavorable chemotherapy or immune checkpoint blockade (ICB) response [35].

GICs represent a highly heterogeneous group of tumors with distinct anatomical, molecular, and immunological characteristics, which directly shape the composition and spatial organization of the TME [18]. Unlike many other solid tumors, GICs arise across multiple organs, including the esophagus, stomach, pancreas, liver, and colorectum, each characterized by unique inflammatory contexts and stromal architecture [17]. This diversity is further amplified by the presence of specific molecular subtypes, such as MSI and MSS, which are particularly relevant in CRC and are associated with markedly different immune landscapes, ranging from highly inflamed, TIL-rich tumors to immune-desert phenotypes [19]. In addition, the gut microbiome plays a critical and relatively unique role in shaping the TME in GICs, especially in CRC, where microbial composition influences immune infiltration, local inflammation, and tumor–immune interactions [20,21]. Furthermore, certain GICs, such as pancreatic cancer, are characterized by a prominent desmoplastic stroma, which creates a dense and immunosuppressive microenvironment that significantly differs from other tumor types [22]. These features collectively introduce substantial variability in tissue architecture, including the presence of mucin, necrosis, and heterogeneous inflammatory patterns, which complicate histopathological assessment. Consequently, they represent unique challenges for AI-based digital pathology, requiring robust computational models capable of handling high spatial heterogeneity and complex tissue organization [23].

### 3.2. Histopathological Markers of the TME in Routine GIC Pathology

The TME in GICs can be characterized in routine histopathological practice through a range of markers reflecting stromal composition, immune infiltration, vascularization, and tumor–stroma interactions, as elaborated in subsequent sections.

- Tumor budding

TB, considered to have high prognostic value, is an independent prognostic factor, strongly associated with EMT, and various factors in TME, which are discussed in greater detail in Section 3 [8,9].

- Tumor-infiltrating lymphocytes

TILs defining all lymphocytic populations infiltrating the tumor tissue are associated with good outcomes from treatment with ICIs, which is reviewed comprehensively in Section 4 [10].

- Tertiary lymphoid structures

TLSs are defined as clusters of immune cells found in non-lymphoid tissues with structural and functional characteristics resembling lymph nodes and the spleen. The presence of dense lymphoid aggregates at the invasive margin (IM) of tumors with a lack of classic germinal centers is considered unorganized as lymphoid infiltrates are referred to as a Crohn’s-like lymphoid reaction (CLR) [6]. Both TLSs and CLRs are further discussed in Section 5.

- Tumor–stroma ratio

Tumor–stroma ratio (TSR) is the proportion of tumor cells in the stroma and is considered to be a novel and practical prognostic predictor in GICs such as esophageal cancer or colon cancer, where low TSR is associated with a poor prognosis. TSR is an important biomarker due to the fact that in tumor tissue the stroma is a major part of the TME that could accelerate tumor progression [36,37].

- Others

Another crucial TME component, TAMs, are a subpopulation of macrophages present in TMEs which both inhibit and promote malignancy. Proinflammatory M1-type TAMs enhance antitumor immunity by providing a secretion of cytokines including interleukin-12 (IL-12) and tumor necrosis factor alpha. M-2 type TAMs enhance tumor progression by facilitating angiogenesis, metastasis, and immunosuppression. Moreover, M2-type TAMs in hypoxic regions remodel the ECM by secreting a vascular endothelial growth factor (VEGF), transforming growth factor-beta and matrix metalloproteinase, which promote angiogenesis, cell migration, and immune response suppression. The dual function of TAMs is regulated by different cytokines, various signaling pathways, and metabolic cues within the TME. TAMs interact intensively with other immune system cells, secreting interleukin-10 (IL-10) and transforming growth factor-beta to inhibit the activity of cytotoxic T lymphocytes and natural killer cells (NK cells), and promoting the expansion of regulatory T lymphocytes (Tregs), which help tumors escape immune control [38,39].

Importantly, CAFs, which are present in tumor TMEs, are diverse cells with numerous roles within the TME including enhancing tumor promotion and inflammation, as well as the maintenance and reshaping of the ECM [40]. CAFs are described as a highly heterogeneous and functionally diverse population of mesenchymal cells whose functions are likely distinct from those of normal tissue-resident fibroblasts. CAFs synthesize cytokines, chemokines, metabolites, enzymes, and ECM components, which directly influence the growth and behavior of cancer cells. Furthermore, through the production of ECM components and matrix remodeling enzymes, CAFs alter the structure and mechanical properties of the TME and influence the proliferation and migration of cancer cells by secreting growth factors and paracrine signals. In addition, they modulate angiogenesis and immune infiltration [41].

The rest of this paper describes in detail three key components of TME—TB, TILs, and TLSs—as they are among the best characterized parameters in GICs. Their significant prognostic and, in some studies, predictive value has been demonstrated, while their unambiguous morphological features make them particularly suitable for standardized assessment in digital pathology and AI-based analyses.

### 3.3. The Clinical and Therapeutic Relevance of the TME in GICs and a Rationale for This Review

A TME influences both tumor progression and response to therapy, primarily via immunosuppression, which inhibits the effectiveness of therapy, including immunotherapy, chemotherapy, and monoclonal antibodies. The TME can weaken therapeutic efficacy through immunosuppressive mechanisms and operate as a therapeutic barrier. Therefore, the modulation of the TME is essential for optimizing treatment outcomes [5,32,42].

High-grade TB is a negative prognostic factor in GICs and is associated with more aggressive tumor characteristics, including a lower degree of histological differentiation, the presence of lymphovascular invasion, and the presence of lymph node metastasis, which contributes to shorter overall survival (OS) [43].

TIL infiltration in gastric cancer (GC) has potential prognostic value, and changes in TILs reflect the strength of the immune response to the tumor. A high level of TILs may be associated with a more favorable disease course and a better response to immunotherapy [32]. However, there is currently no consensus on the clear impact of low TILs on prognosis in GC, and research results are heterogeneous and difficult to compare [44].

High TLS density in tumors is associated with better patient survival and a lower risk of disease recurrence. Mature TLSs, containing specialized T and B zones and high endothelial venules (HEV) activity, promote an effective local immune response, which translates into favorable clinical outcomes. TLSs act as local immune centers, supporting the activation and proliferation of cytotoxic T and B cells, which strengthen the antitumor response of the immune system. The presence of TLSs is therefore considered a positive prognostic marker in gastrointestinal cancers [45].

TSR can serve as a prognostic indicator of TME advancement; a higher TSR correlates with a more aggressive tumor and a poorer prognosis, as a greater number of stromal components may promote tumor proliferation and invasion [44].

Tumors with a high mutational burden and increased T-cell infiltration are more likely to respond to ICB therapy, as a greater neoantigen load promotes T-cell activation. Therefore, there is an undeniable correlation between inflammation and TME and better therapeutic efficacy. Microsatellite Instability-High Colorectal Cancer (MSI-H CRC) is commonly described as an inflamed TME with a high cytolytic activity of T lymphocytes and the presence of structures such as TLSs, which support an active immune response. This phenotype is associated with improved survival and an effective response to ICIs. Conversely, immune-desert TMEs with a low number of infiltrating T lymphocytes have active mechanisms blocking the migration of T lymphocytes into the tumor which leads to a poor response to immunotherapy. Specifically, subtypes such as Consensus Molecular Subtypes 2 and 3 are characterized by low TIL cytolytic activity, immunosuppressive mechanisms, and poorer response to immunotherapy [5,42].

Given the growing evidence on TME markers in gastrointestinal cancers and the rapid development of digital pathology and AI, there is a need to synthesize data on those histopathological features that are both clinically relevant and technically amenable to automated quantification. In the following sections, we therefore focus on tumor budding, TILs and TLSs as representative TME biomarkers in GICs.

**Figure 1:**
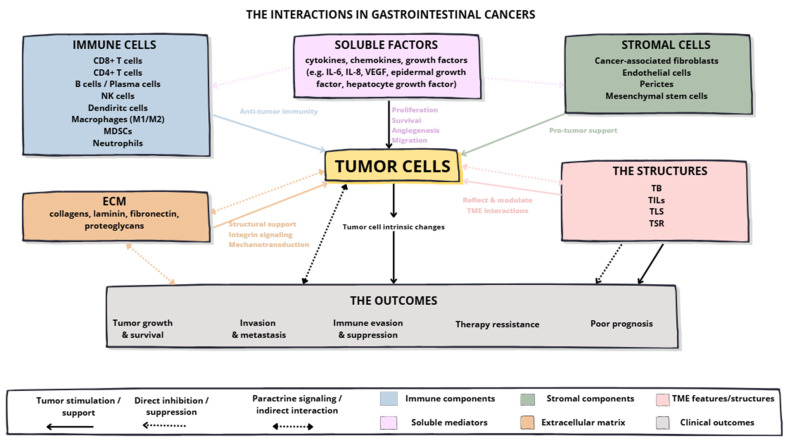
The interactions in GIC TMEs.

A schematic overview of the key interactions within the TME in GICs is presented in Figure 1.

## 4. Tumor Budding: Definition, Clinical Role, and AI-Based Evaluation

### 4.1. Biological Definition

TB is a manifestation of dissociative growth and cellular plasticity at the leading edge of solid tumors. It is morphologically characterized as the presence of individual detached tumor cells or small clusters (<5 cells) located at the invasive tumor front or within the tumor stroma [24,46]. It represents the histological surrogate of EMT. During this process, polarized epithelial cells undergo molecular reprogramming to acquire a mesenchymal phenotype, losing cell to cell adhesion and enhancing the migratory capacity. EMT is often marked by the downregulation of epithelial markers, such as E-cadherin, and the upregulation of mesenchymal drivers, allowing these buds to detach from the primary tumor mass and infiltrate the surrounding tissue [24,47,48].

Tumor buds are regarded as the precursors to lymphovascular invasion and distant metastasis. Their presence reflects a tumor’s inherent ability to penetrate the extracellular matrix and enter the systemic circulation [24,49]. Moreover, molecular TB has been linked to specific drivers of malignancy, such as *KRAS* mutations and MSS status, further underscoring its role as a biomarker of distinct, aggressive biological phenotypes [46,48]. TB by integrating the EMT, stromal permissiveness, and immune subversion, has a great potential to predict a high risk of recurrence and diminish disease-free survival (DFS) [49,50,51].

### 4.2. Clinical Significance in GIC

Extensive evidence supports TB as an independent prognostic factor, especially in CRC, where it is incorporated into clinical guidelines. High tumor budding in CRC is typically defined as the presence of ≥10 buds in a 20× objective field and has been confirmed as a robust predictor of adverse outcomes. In population-based cohorts, high TB is significantly associated with an advanced pathologic stage, MSS, *KRAS* mutations, AND is independently associated with a worse cancer-specific survival rate [46]. In stage II and III colon cancer, TB is a critical biomarker for risk stratification. Patients with high-grade budding exhibit shorter time to recurrence, with 24-month freedom from recurrence rates dropping to 69% in comparison to 92% in low-grade budding cases [49]. Moreover, in stage III rectal carcinoma, the intensity of budding at the IM is an independent variable for the DFS. Integrating TB scores with traditional N-stage parameters provides a more precise prognostic stratification than the American Joint Committee on Cancer’s nodal staging system alone, allowing for a more granular identification of tumors with higher malignancy potential [50].

Emerging studies indicate similar significance in GC and pancreatic cancers. In GC, data from meta-analysis involving over 2100 patients revealed that high-grade TB is associated with aggressive clinicopathological parameters, including advanced tumor stage, lymphovascular invasion, and lymph node metastasis [24]. A comprehensive whole-slide imaging (WSI) analysis of 75 GC cases revealed that infiltrative growth patterns in both the marginal zone and basal zone were identified as independent risk factors for TB. The analysis also confirmed that TB and TB grade, along with the American Joint Committee on Cancer stage, lymph node metastasis, and pT stage, influenced prognosis in GC patients [52]. A 2024 meta-analysis of 57 studies demonstrated that high-grade TB in both gastric and pancreatic adenocarcinoma was associated with worse OS [53]. A 2025 Turkish cohort study found that TB was an independent prognostic factor with a median survival rate of 7.03 months in patients with tumor budding compared to 21.7 months in patients without it [51]. Additionally, TB has a prognostic value in patients receiving neoadjuvant therapy [48,54].

Recent advancements in AI and digital pathology have evaluated the clinical significance of TB in GICs. Deep learning convolutional neural networks (CNNs) have been developed to automate bud detection, achieving prognostic values comparable to manual visual assessment [24]. Current AI platforms demonstrate high sensitivity and specificity in identifying TB and its relationship with the TME, which may provide a valuable additional examination into the pathology reports [25].

### 4.3. Tumor Budding, Tumor–Stroma Ratio, and Desmoplastic Reaction as “Invasive Front Signature”

TB, TSR, and DR constitute an “invasive front signature,” capturing the combined behavior of invading tumor cells and the associated stromal remodeling, a multidimensional indicator of tumor aggressiveness at the leading edge. Rather than viewing these as isolated histological markers, recent evidence suggests they represent a coordinated snapshot of how tumor cells decouple from the primary mass and how the surrounding stroma is reprogrammed to facilitate that invasion [14,25,55].

TSR quantifies the relative proportion of stromal tissue versus tumor epithelium within the tumor mass, which is typically assessed at the invasive front. Tumors are classified as stroma-high, where stromal area is above 50%, or stroma-low, where stromal area is below 50%, based on a visual estimation on hematoxylin and eosin (H&E) stained sections [9]. Recent evidence from a multicenter cohort of 497 stage II colon cancer patients demonstrates that the prognostic effect of TSR is specific to assessment at the deepest invasive front and loses significance as the examination area expands. Patients with stroma-high tumors exhibited significantly shorter 5-year time to recurrence, recurrence-free survival, and OS in comparison to stroma-low tumors [56].

Desmoplastic reaction (DR) represents the fibrous tissue reaction surrounding tumor cells and reflects the quality and maturity of the tumor stroma. DR is histologically classified into three categories based on the products of activated fibroblasts: mature DR (characterized by keloid-like collagen bundles with minimal myxoid stroma), intermediate DR (mixed features of mature and immature stroma), immature DR (myxoid, loosely organized stroma with abundant activated fibroblasts) [57,58,59]. Across multiple international cohorts, immature DR is the most significant and independent prognostic feature for disease-specific survival in stage II CRC. Moreover, immature DR is strictly associated with high-grade TB, suggesting a close biological relationship between the invasive cellular phenotype and the stromal microenvironment [58,59,60,61].

The combination of TB, TSR, and DR provides a superior prognostic stratification, supporting the concept of an integrated “invasive front signature”. The synergistic prognostic value of combining these markers underscores the importance of assessing the invasive front as an integrated biological unit.

### 4.4. Limitations of Manual Evaluation

Despite the clinical utility of TB as a prognostic marker, it has been hindered from routine pathology reports by several methodological challenges. Manual assessment remains fundamentally observer-dependent, leading to interobserver variability even among experienced pathologists. This variability is often compounded by the lack of the universally established histologic cutoffs for a high-grade budding and the heterogeneity of the study populations, which may lead to conflicting results regarding its prognostic significance [43,46].

A primary technical hurdle in manual evaluation is the identification of the invasive front. Pathologists must visually scan the entire IM to select a single objective field for the quantification process, which is both time-consuming and prone to selection bias [24]. The inherent tumor heterogeneity across a single slide means that manual methods may fail to capture the true biological behavior of the malignancy [50].

Moreover, visual quantification has a practical barrier in high-volume clinical settings. The requirement for the meticulous counting of single cells or small clusters in a specified area is both inefficient and susceptible to errors from exhaustion [24,25]. These limitations underscore the necessity for more objective, reproducible, and automated quantification methods, e.g., using AI to facilitate the standardized reporting of TB in GICs [25].

### 4.5. AI-Driven Detection and Quantification

The limitations of the manual tumor budding assessment have driven the development of AI-based automated detection and quantification systems that promise to standardize TB assessment, reduce interobserver variability, and facilitate integration into clinical trials [24,62,63]. Deep learning models, including CNNs, such as Faster R-CNN, Mask R-CNN, U-Net, RetinaNet, and transformer-based approaches have achieved high accuracy in identifying and counting tumor buds in WSI. The most advanced systems include fully automated pipelines that replicate the entire International Tumor Budding Consensus Conference (ITBCC) workflow. This process includes deep learning-based segmentation for tumor border identification, individual bud detection, characterization based on cell count (1–4 cells), and density map generation across the entire invasive front. Automated hotspot selection of the 0.785 mm^2^ area with the highest budding density and automated counting with three-tier grading (BD0: 0 buds, BD1: 1–4 buds, BD2: 5–9 buds, BD3: ≥10 buds) provide reproducibility across different clinical institutions that would be difficult to achieve manually [24,25,60,63,64].

To maximize performance despite limited annotated data, advanced strategies such as semi-supervised and weakly supervised models are utilized. Semi-supervised learning leverages both labeled and unlabeled data to achieve prognostic values comparable to visual assessment and remaining objective quantification [24]. Weakly supervised models utilizing Bayesian Multiple Instance Learning have enhanced the generalizability of these tools, achieving precision of 0.94 and high stability during cross-validation. These advancements ensure that AI performance is increasingly comparable to that of expert gastrointestinal pathologists [25,57]. Moreover, a landmark study validated a fully automated pipeline against a panel of five expert pathologists on a cohort of 981 CRC patients. The results of the AI system at detecting individual tumor buds were similar to the pathologists’, while AI maintained independent prognostic value in both univariate and multivariate analysis [62]. Furthermore, strong correlations were observed between the proportion of positive lymph nodes and algorithm measures of TB compared to manual counts. Beyond simple bud counting, AI-based systems also enable the extraction of novel metrics that capture biological nuances which are inaccessible through conventional visual assessment. These include tumor bud density, tumor bud dispersion, intratumoral heterogeneity, and tumor–stroma interface features [24].

Emerging solutions and future perspectives include the development of pathology foundation models pretrained on massive diverse databases with multimodal learning integrating histopathology, radiology, genomics, and clinical data. Such integrated approaches offer a more comprehensive, personalized snapshot of tumor aggressiveness and patient prognosis.

### 4.6. Limitations and Challenges

Despite robust evidence supporting TB as an adverse prognostic marker, several limitations temper its translation onto standardized AI-assisted reporting. The TB evidence base is heterogenous: Studies differ in TB definitions, staining, cut-offs, and whether intratumoral or peritumoral buds are scored. This leads to variable effects and makes cross-study comparison harder [43]. Meta-analyses in non-CRC GICs report consistent associations between high TB and poor survival but also highlight substantial statistical heterogeneity and a lack of large, uniformly assessed, organ-specific cohorts, which constrain guideline adoption [43]. In addition, many TB studies are single-center, retrospective, and focus on selected histologic subtypes, which may overestimate prognostic value and limit generalizability.

AI-driven TB detection also faces technical and methodological challenges. Most deep learning pipelines for TB are trained on relatively small or restricted datasets, risking overfitting to local staining, scanners, or cases [43,65,66]. Systematic reviews of AI for TB or TMEs and digital pathology broadly show very high reported accuracies, but also emphasize heterogeneous performance metrics, small samples, and frequent high or unclear risk of bias [53,66]. External validation is either absent or often limited to one additional cohort, mirroring wider computational pathology and oncology literature where model performance commonly drops on independent data due to cohort differences [24,67,68,69,70,71].

Moreover, AI models may inherit or amplify dataset bias and imbalance, for example through the underrepresentation of certain tumor subtypes, stages, or demographic groups or through site-specific artefacts in large repositories such as The Cancer Genome Atlas (TCGA) [24,71,72]. These issues, put together with limited transparency, incomplete reporting, and variability in validation practices currently restrict AI-based TB quantification to a promising research and decision-support role rather than a fully developed, universal clinical tool [24,53,66,70].

## 5. Tumor-Infiltrating Lymphocytes: Immunological Relevance and Digital Analysis

TILs are essential immunological biomarkers that reflect the interactions between the host immune system and the TME [73].

A 2018 study by Lee et al. [74] has shown that TIL density, particularly cytotoxic CD8^+^ T cells, is related to clinical outcomes and responses to immunotherapy in patients with GIC. The CD8^+^ T lymphocytes are important mediators of the host immune response against cancer cells. TIL infiltration affects tumor growth control, disease progression, and metastatic potential. TIL levels might also reflect the response to cytotoxic therapies, such as chemotherapy and radiotherapy. Accordingly, elevated TILs, including CD8^+^ lymphocytes, might be prognostic markers in GC [74].

Recent developments in digital pathology and AI have enabled more accurate and objective assessment of immune infiltration within tumors. AI-based approaches allow the automated detection and phenotypic categorization of TILs. These technologies may facilitate high-resolution spatial analysis and more consistent immune profiling. However, their clinical utility still depends on external validation, standardized pipelines, and a demonstration of added value over established histopathological and molecular markers. They are also increasingly applied to guide clinical decision-making and the selection of therapeutics. In addition, computational TIL analysis and standardized frameworks, such as the Immunoscore, are now recognized in routine oncologic pathology and precision immuno-oncology [75]. Importantly, the dynamics of T-cell responses, including activation, exhaustion, and checkpoint regulation, play a crucial role in shaping the effectiveness of antitumor immunity [76].

### 5.1. Biological and Immunological Role

Recent advances in cancer immunology have highlighted the dynamic and interactions between immune and tumor cells, which play an important role in tumor progression [77,78]. Immunotherapy may be effective in tumors with a high mutational burden [79,80]. This feature is observed in a subset of GICs, therefore understanding immune response mechanisms within the TME is crucial for the development of effective immunotherapeutic strategies [26,28,73,74,81].

TILs have a twofold role. On the one hand, they can inhibit tumor growth by directly killing malignant cells or restraining their proliferation. On the other hand, they may promote tumor progression by selecting more resistant tumor cell clones or by creating conditions favorable to tumorigenesis [73]. It is observed that a T-cell-inflamed TME is characterized by the presence of a large number of immune cells surrounding the tumor cells and the tumors having such microenvironments shows better response to the immunotherapies [74].

Cytotoxic CD8^+^ T lymphocytes represent the main effector cells of antitumor immunity. Cytotoxic T lymphocytes recognize tumor antigens and eliminate malignant cells. Their mechanism is the release of cytotoxic mediators such as perforin, granzymes, and cytokines. Their activity can also involve ferroptosis and pyroptosis. CD4^+^ T helper cells regulate immune responses and differentiate into several functional subsets, including Th1, Th2, Th17, and Tregs. Th1 promotes the activation and expansion of cytotoxic T lymphocytes. In contrast, Th2 and Th17 cells produce immunosuppressive cytokines that may promote tumor progression. Tregs, characterized by FOXP3 expression, suppress antitumor immunity by limiting the activation and proliferation of effector T cells and by competing for essential cytokines such as IL-2 [73].

Immunotherapy has recently become an important treatment strategy for patients with CRC. However, the clinical response to these therapies varies substantially among individuals. This variability highlights the importance of a detailed characterization of the TME, which plays a critical role in modulating anti-tumor immune responses and may influence treatment outcomes [14].

### 5.2. Prognostic and Predictive Value in GIC

A systematic review and meta-analysis of CRC demonstrated that the density and distribution of CD3^+^, CD8^+^, and CD45RO^+^ T lymphocytes in the tumor center and invasive margin are stronger prognostic factors for patient survival than the TNM system [81]. This finding is consistent with their biological functions. CD8^+^ cytotoxic T lymphocytes play a key role in the direct elimination of tumor cells, while CD45RO expression indicates a memory T-cell phenotype, suggesting prior recognition of tumor antigens by the immune system [81]. In GC, high CD3^+^ infiltration is generally associated with improved DFS, although its relationship with OS may vary depending on tumor location [73].

TILs are composed of a mix of lymphocytes such as CD3^+^, CD8^+^, CD4^+^, and FOXP3^+^ cells. These immune cells reflect the host’s immune response to the tumor and may contribute to tumor control and influence response to therapy [82]. High densities of TILs, particularly CD8^+^ T cells, are associated with a favorable prognosis in several GIC, including GC, CRCl, and pancreatic cancer. Increased infiltration of CD3^+^, CD4^+^, CD8^+^, and CD45RO^+^ lymphocytes correlates with longer survival, supporting an important role of T-cell-mediated immune responses in tumor control. CD8^+^ lymphocytes appear to show the most consistent association with improved survival across studies [63,64,65,66,67,68,69,70,71,72,73,74,75,81].

In contrast, the prognostic role of FOXP3^+^ TILs remains controversial. In GC, high FOXP3^+^ infiltration has been associated with poorer survival in some studies, whereas others found no significant association with the prognosis [73,74]. In CRC, higher FOXP3^+^ cell density has in some cases been linked to improved survival, suggesting a context-dependent effect [81].

In pancreatic cancer, the prognostic role of TILs has also been reported. Higher densities of CD3^+^ and CD8^+^ lymphocytes are associated with improved survival, whereas FOXP3^+^ cells tend to indicate poorer outcomes. Notably, pancreatic tumors are characterized by a strongly immunosuppressive microenvironment, which may limit effective antitumor responses [75].

In addition to their prognostic value, TILs may also have predictive significance. In locally advanced rectal cancer treated with neoadjuvant chemoradiotherapy, higher CD8^+^ T-cell infiltration has been associated with better tumor regression grade and improved long-term outcomes [81,83].

Tumor infiltration phenotypes describe how immune cells are spatially distributed in relation to tumor cells within the TME. In CRC, two main patterns have been identified. The inflamed phenotype is characterized by the close proximity of immune cells to tumor cells, suggesting active immune engagement, whereas the immune-excluded phenotype refers to situations in which immune cells remain separated from tumor nests, indicating barriers that limit effective infiltration. These patterns may help to better understand tumor–immune interactions. However, their prognostic and predictive value in CRC has not yet been clearly established [14].

### 5.3. Challenges of Manual Tumor-Infiltrating Lymphocytes Assessment

The manual assessment of TILs in histopathology has several limitations. Distinguishing lymphocytes from stromal nuclei on H&E-stained sections can be difficult, especially in areas of high cellular density. In such regions, nuclear morphology alone is often insufficient for reliable identification. This can reduce the accuracy and reproducibility of TIL evaluation [82,84].

This type of assessment is inherently subjective and prone to both inter- and intra-observer variability. Differences in experience, interpretation, and applied scoring systems contribute to inconsistencies across studies and clinical settings [82,84]. The lack of universally accepted and standardized criteria for TIL quantification further limits comparability between cohorts [82].

The quantitative evaluation of TILs in routine histopathology remains challenging. It is time-consuming and labor-intensive. It is also difficult to standardize, especially when large tissue areas or whole-slide sections are assessed. As a result, microscopic evaluation is often semi-quantitative and based on visual estimation rather than precise measurement [77,82].

Another limitation is the spatial heterogeneity of TIL distribution within tumors. Immune cell density can vary across regions, including the tumor center, invasive margin, and surrounding stroma. As a result, hotspot-based or area-restricted analyses may not reflect the overall immune landscape and can introduce sampling bias [26,81].

Assessment of immune cell density using immunohistochemical (IHC) staining is resource intensive. It requires the evaluation of multiple microscopic fields and a considerable amount of time. Although IHC staining allows for more precise identification of immune cell subtypes, its routine use is limited by cost and the need for specialized expertise [27,77,82].

In contrast, H&E-based assessment remains widely used in both research and clinical practice. It is simple, accessible, and cost-effective. This is particularly important in low- and middle-income settings, where advanced digital or molecular techniques may not be readily available [26,77].

Overall, manual TIL evaluation is limited by subjectivity and reduced reproducibility. It also fails to capture complex spatial relationships within the TME. These limitations highlight the need for more objective and standardized approaches to immune profiling [26,27,30].

### 5.4. Immunoscore as a Standardized Metric, Naturally Suited to AI

The prognostic importance of TILs was first shown in CRC studies. Subpopulations of CD3^+^ T cells and cytotoxic (CD8^+^) and memory (CD45RO^+^) have been shown to be important. Results from these studies gave rise to the Immunoscore scale [77,81]. This system aims to assess the extent of immune cell infiltration into the tumor core and IM. Subsequently, Immunoscore has been confirmed as a prognostic tool that surpasses the American Joint Committee on Cancer TNM classification in predicting long-term treatment outcomes for patients. A large international study confirmed Immunoscore inter-center reproducibility and the fact that it could be considered a reliable predictor of time to recurrence independent of other conventional prognostic parameters such as age, T stage, N stage, and MSI [81].

The main advantage of AI for immune profiling is that some biomarkers are intrinsically less complicated to standardize and quantify computationally than traditional visual TIL assessment. Immunoscore offers a strong reference because this method depends on the highly defined anatomical regions and quantitative assessment of CD3^+^ and CD8^+^ lymphocyte density in both the tumor core and IM. In the work of Dienstmann et al., digital pathology was deployed to automatically detect tumor locations, measure immune-cell densities, and compute Immunoscore, which included a more compact version, Immunoscore Immune-Checkpoint (ISIC). Most interestingly, ISIC-high tumors were more frequent than IS-high tumors and had a more immunogenic TME and mutational load. This suggests that computational methods may extend and further refine standardized, spatially defined immune metrics [78].

Recent advances in deep learning reinforce this idea. AI techniques were used in a study that analyzed routine histopathological slides of WSI of CRC in a recent study. The new model automatically recognized tumor areas. It also detected TILs and measured both their density and spatial distribution, and the entire process was fully automated. Based on this characteristic information, patients were divided into distinct risk groups. Patients classified as high-risk had worse OS than low-risk patients. This supports the prognostic importance of AI-based immune features. Crucially, the analysis was conducted on basic H&E-stained slides without further IHC analysis. We conclude that computational models are able to capture spatial immune patterns meaningfully and reliably. These findings reinforce that immune metrics that are structured, such as Immunoscore are compatible with AI-based analysis [85].

Immunoscore has been extended to transcriptomic data beyond image-based approaches. In a pan-cancer real-world cohort of 522 patients treated with ICIs, a CD3/CD8-based Immunoscore estimated by CIBERSORTx related to OS. This indicates that the immune measure based on T-cell infiltration can be converted into computational frameworks [79].

However, the analysis performed on nivolumab patients with metastatic pMMR/MSS colorectal cancer showed that the Immunoscore Immune-Checkpoint (IS-IC) did not precisely predict response to treatment. Only a small number of patients found lasting benefits. Treatment outcomes were particularly dismal in those with liver metastases. These results are a reminder that good immune metrics are not universalizable from the point of view of clinical utility even when they are biologically-established and well-designed [80].

### 5.5. AI-Based Tumor-Infiltrating Lymphocytes Quantification

Advances in digital pathology and AI have transformed histopathological image analysis from a subjective and observer-dependent process into a scalable and quantitative approach, particularly in CRC and other gastrointestinal malignancies. The increasing use of WSI has enabled the application of deep learning algorithms that extract high-dimensional features directly from digitized tissue sections [27].

CNNs are widely applied in histopathological image analysis and perform well in tissue classification, cell detection, and pattern recognition. In CRC, these models allow the identification of TME features that are not consistently detectable by visual assessment alone, including subtle immune infiltration patterns and stromal–tumor interactions [29]. Deep learning-derived features have also been linked to patient survival, providing prognostic information beyond conventional clinicopathological parameters such as TNM staging [86].

AI-based platforms enable reproducible analysis of large histopathological datasets, which is particularly relevant in TIL assessments. Manual evaluation of TILs remains prone to inter-observer variability, especially across CRC and breast cancer specimens, where scoring systems are not uniformly applied [82,84]. In this context, AI-based quantification improves standardization and allows for a more objective assessment of immune infiltration. Integration of digital pathology with clinical and molecular data further supports the development of composite biomarkers in CRC and other GI cancers, including those related to Immunoscore-based stratification [77].

Automated detection and quantification of TILs has been extensively investigated in CRC cohorts. Weakly supervised deep learning approaches allow analysis of WSI without the need for exhaustive manual annotations, enabling scalable immune profiling in large patient populations [87]. The study by Saltz et al. (2018) [26] demonstrated that TILs are spatially heterogeneous across tumors, including CRC, and can be mapped at high resolution using CNN-based models. These spatial maps were strongly associated with gene expression profiles and immune-related molecular signatures [26].

Notably, in CRC, the spatial organization of TILs appears to be more strongly associated with patient outcomes than their absolute density. This observation supports the concept that immune architecture within the TME plays a critical role in tumor behavior and prognosis [81]. However, accurate quantification remains challenging in regions of dense immune infiltration, where overlapping lymphocytes may lead to segmentation errors and the underestimation of cell counts [26].

Recent developments have focused on the higher-order spatial analysis of immune infiltration, particularly in GIC. AI-based models can identify complex spatial patterns, including immune cell clustering and tumor–immune interfaces, which are not easily captured by conventional histopathology. These features have been shown to correlate with clinical outcomes in pan-GIC cohorts, suggesting that spatial immune organization provides additional prognostic value beyond simple TIL density [88].

Models based on interpretable, handcrafted features can capture the structural organization of the TME, including spatial interactions between TILs and adjacent tumor nuclei. These patterns have been shown to provide independent prognostic information across several GICs, such as gastric, colorectal, pancreatic, and liver cancer, sometimes outperforming conventional clinicopathological variables. In general, low-risk tumors tend to show a higher density of TILs closely interacting with tumor cells, suggesting an active anti-tumor immune response, whereas high-risk tumors are characterized by sparse and more disorganized immune infiltration. In contrast to many deep learning approaches, this model remains interpretable, as it is based on graph-derived and morphologic features. This makes it easier to understand tumor–immune interactions and supports clinically relevant risk stratification, for example in patients with MSS stage II CRC.

These findings highlight the limitations of traditional manual assessment, which often fail to capture complex spatial relationships within the TME. However, computational approaches also have their drawbacks. Their performance can be affected by image quality and staining variability, and they typically require large, well-annotated datasets. Moreover, most studies are retrospective, which may limit generalizability, and biopsy-based analyses may not fully reflect tumor heterogeneity. Despite these limitations, such methods provide a more objective, reproducible, and scalable alternative to manual evaluation [88].

Computational pathology enables the identification of complex spatial patterns and tumor–immune interactions that are not readily detectable by conventional microscopic evaluation. It extends the diagnostic and prognostic value of TIL assessment beyond simple cell quantification [84].

More advanced computational pathology approaches enable the inference of molecular and phenotypic information directly from routine histopathological images. The ROSIE model, for example, generates silico multiplex immunofluorescence from standard H&E slides and has been applied to multiple tumor types, including CRC [89]. This approach allows the detailed characterization of immune cell populations and their spatial relationships with tumor cells. As it relies on widely available H&E preparations, it remains scalable and applicable in large clinical datasets while extending the analytical depth of routine pathology [27,28].

Clinical applications of AI-assisted immune profiling are particularly evident in CRC and rectal cancer. Quantitative and spatial features of TILs derived from histopathology slides have been shown to predict response to chemoradiotherapy in both clinical trial and real-world settings [83]. Similarly, AI-based analyses in CRC can identify patterns associated with prognosis, treatment response, and molecular subtypes, supporting their role in precision oncology [29].

A further challenge is the laborious task of evaluating immune cell counts by immunohistochemistry. This work assessed the prognostic accuracy of immune cell densities in the TME using AI-driven whole-slide H&E staining of WSI. Three CRC cohorts (one all-stage and two cohorts of patients with locally advanced rectal cancer treated with neoadjuvant chemoradiotherapy) were included as study subjects. Tumor samples were then identified by a tissue classifier (EfficientNet) using an AI framework; whereby an object detection model (YOLO) was fitted to automatically detect lymphocytes, macrophages, and mitotic figures in the whole tumor, and identified tumor regions. Increased TIL density in combination with decreased TAM density were associated with greater DFS and OS, where DNA mutation status also influenced TILs, and post treatment increased TIL levels were associated with more favorable outcomes. These results illustrate the challenges of assessment by hand as a qualitative assessment and highlight the benefits of AI, which facilitates more objective, reproducible, and scalable assessment. A whole-slide analysis might still produce lower average cell densities than hotspot-based approaches, and the differentiation of immune cell subtypes presented by H&E images is still limited without IHC markers. Nevertheless, total immune cell density is still prognostic, and the described method is considered in practice and feasible, especially for resource-limited environments. Study limitations included limitations to quality control in large cohorts and that it may underestimate tumor heterogeneity in biopsy samples [83].

Patients who respond to immunotherapy, including those with CRC, often exhibit higher densities and specific spatial distributions of CD8^+^ T cells, reflecting active anti-tumor immune responses. However, these highly infiltrated regions remain challenging for automated image analysis, as tightly packed lymphocytes can reduce segmentation accuracy and lead to false-negative detection [30,76].

Accurate assessment of immune cell density using IHC staining is time-consuming and labor-intensive. It requires manual analysis of numerous fields of view. This limits its use in routine pathomorphological diagnosis. The advancement of digital pathology and AI has facilitated the automated, cell-level quantification and phenotypic classification of TILs. These technological advancements support high-resolution spatial analysis and standardized immune profiling [74].

Importantly, most available evidence on AI-based TIL quantification originates from retrospective studies, predominantly in CRC cohorts. This may limit generalizability across tumor types and highlight the need for prospective validation and standardized analytical pipelines before widespread clinical implementation [27,28,29].

Nevertheless, methodological and biological challenges still impede AI-based TIL quantification. Most of the available models were developed and validated mostly in cohorts of colorectal cancer, limiting their direct generalizability to gastric, pancreatic, hepatobiliary and esophageal cancers [27,28,29]. Furthermore, H&E-based approaches are unable to reliably differentiate between lymphocyte subpopulations, activation states or exhausted phenotypes without immunohistochemistry or multiplexed methods, which may limit their biological interpretability [26,83]. Additional limitations are staining and scanner variability, tumor sampling differences, segmentation errors in densely infiltrated regions, and a lack of independent external validation in many cases [26,30,83]. Thus, AI-derived TIL metrics should be currently viewed as promising spatial biomarkers and decision-support tools rather than fully standardized clinical parameters.

## 6. Tertiary Lymphoid Structures: Biology, Clinical Relevance, and Digital Pathology Approaches

### 6.1. Overview of Tertiary Lymphoid Structures

TLSs are ectopic structures that form in non-lymphoid tissues in response to chronic inflammation, including neoplastic processes. They show numerous similarities to secondary lymphoid organs, such as lymph nodes; however, unlike these, they are not developmentally programmed in advance but develop locally in response to environmental stimulations [90,91].

The architecture of a TLS is highly organized and includes distinct functional zones: Clusters of B cells, often containing proliferation centers, and areas rich in T cells and dendritic cells. This organization enables the effective presentation of the antigen and the initiation of an adaptive immune response. Moreover, it promotes the maturation of the humoral response through processes such as somatic hypermutation and clonal selection [92,93].

Specialized blood vessel HEVs play a key role in TLS function, facilitating the influx of lymphocytes from the blood to the site of the immune response. The presence of HEVs indicates the active recruitment of immune cells and is one of the markers of functional TLS maturity [92,93].

The formation of a TLS is a complex process regulated by chemokines such as CXCL13, CCL19, and CCL21, which are responsible for the recruitment and spatial organization of lymphocytes. Stromal cells also play a significant role, not only providing a structural scaffold but also producing signaling factors that sustain TLS function [90,91].

The degree of TLS organization can vary from simple clusters of lymphocytes to fully developed structures containing proliferation centers, which directly translate into their immunological potential [91,93].

### 6.2. Tertiary Lymphoid Structures in GIC

In GIC, TLS are among the best-documented markers of an active immune response. Their presence in CRC, GICs, and pancreatic cancer correlates with a favorable clinical prognosis, which includes both prolonged OS and a reduced risk of disease recurrence [94,95,96].

In CRC, TLSs are most commonly located at the margin of the tumor infiltrate and are associated with high immune activity. Mature structures containing proliferating centers are of particular importance, as they indicate an active humoral response and are strongly associated with a better prognosis [94,97].

In GICs, TLSs also play a prognostic role and are associated with greater infiltration of effector lymphocytes. In contrast, in pancreatic cancer, which is characterized by a strongly immunosuppressive microenvironment, TLSs occur less frequently; however, their presence may indicate a shift in local immunosuppression [91,98].

Another important aspect is the role of TLSs in predicting responses to immunotherapy. It has been demonstrated that the presence of these structures, particularly mature ones, is associated with a better response to ICIs, making them a potential predictive biomarker [99,100].

TLSs provide information that goes beyond traditional markers, such as the number of TILs, because they reflect their functional organization and ability to initiate an effective immune response [95,96].

### 6.3. Crohn’s-like Lymphoid Reaction

CLR is a specific form of immune response observed primarily in CRC, characterized by the presence of localized, nodular lymphocyte aggregates that occur primarily at the margin of the tumor infiltrate and in tissues adjacent to the tumor, such as the muscularis propria and pericolonic adipose tissue [101].

Morphologically, a CLR resembles the changes observed in Crohn’s disease, which is reflected in its name. Lymphocytic aggregates can take various forms ranging from loose clusters of immune cells to more organized structures resembling lymphoid nodules, sometimes containing proliferative centers [101].

Biologically, CLR is considered a component of the host’s adaptive immune response against the tumor. In the early stages of development, T lymphocytes (especially CD4^+^) and antigen-presenting dendritic cells dominate, whereas as the structure matures, B lymphocytes and nodal dendritic cells are recruited, leading to the formation of more organized structures resembling TLS. For this reason, CLRs are often interpreted as a developmental continuum of TLSs ranging from early, loosely organized lymphocyte aggregates to fully functional structures capable of initiating an immune response. Mature forms of CLR can function as local “immune niches” where lymphocyte activation, antigen presentation, and the development of humoral and cellular responses occur [101].

The clinical significance of CLRs has been extensively documented in CRC research. Numerous analyses have shown that the presence of an intense CLR is associated with significantly better prognosis, including prolonged OS and a lower risk of death, independent of classical prognostic factors [97,102].

In particular, it has been shown that tumors characterized by a pronounced presence of CLR have a lower risk of disease progression and a better immune response, suggesting that CLR may serve as a marker of effective tumor growth control by the immune system [97,101].

However, the lack of standardized criteria for assessing CLR remains a significant problem. Various methods for quantifying CLR are used in the literature, based, among other things, on the number, size, or density of lymphocyte aggregates, which leads to significant differences in the interpretation of results and limits the use of CLR as a standardized clinical biomarker [93,102].

In response to these limitations, new approaches are being developed, including methods based on AI, enabling the automatic and objective assessment of CLR density in histopathological images. Preliminary studies indicate that such approaches may improve the accuracy of assessment and enhance the prognostic value of CLR in clinical practices [93].

CLR constitutes a significant component of the TME in CRC, reflecting an active host–immune response. Its presence is associated with a favorable prognosis and may represent a promising prognostic biomarker as well as a potential target for further research on immunotherapy [97,101].

### 6.4. Biological and Clinical Relevance

TLSs play a key role in the local regulation of the immune response, serving as specialized microenvironments that facilitate the initiation and maintenance of the adaptive immune response. These are ectopic aggregates of immune cells that form in non-lymphoid tissues in response to chronic antigenic stimulation and persistent inflammation [103,104].

Unlike secondary lymphoid organs, TLSs do not develop during embryogenesis but arise de novo within tissues affected by pathological processes, such as tumors, chronic infections, or autoimmune diseases [105,106].

These structures are characterized by a complex cellular organization comprising T and B lymphocytes, dendritic cells, and stromal elements resembling those present in lymph nodes. Within TLSs, effective antigen presentation by dendritic cells and T-cell activation occurs, initiating a local immune response. At the same time, TLSs serve as sites for the proliferation and differentiation of B lymphocytes and their transformation into antibody-producing plasma cells, making them functional centers of the humoral response [103,107].

As TLSs mature, they can develop a highly organized structure containing germinal centers, which makes them functionally similar to classical lymphoid organs. In such mature TLSs, key adaptive immune response processes occur, including B-cell clonal selection, immunoglobulin class switching, and antibody affinity maturation [91,108].

Importantly, TLSs play an active role in shaping the TME. Through the local secretion of chemokines and cytokines, they promote the recruitment of effector cells and sustain the antitumor response. It has been demonstrated that the presence of TLSs is associated with the increased infiltration of CD8^+^ T lymphocytes and enhanced cytotoxic responses against tumor cells [106,109].

From a clinical perspective, TLSs have significant prognostic and predictive value. Numerous studies have shown that their presence correlates with a better prognosis and prolonged OS in many solid tumors. Furthermore, TLSs are strongly associated with responses to immunotherapy, particularly ICIs, making them a promising biomarker of treatment efficacy [104,105].

### 6.5. Challenges in Manual Tertiary Lymphoid Structure Assessments

Despite the growing importance of TLSs as a component of the immune microenvironment and a potential biomarker of treatment response, their assessment in routine histopathological practice remains a significant diagnostic challenge. One of the main problems is the lack of clear, universally accepted criteria for identifying TLSs. In pathological practice, it is often difficult to distinguish well-organized TLSs from diffuse, nonspecific inflammatory infiltrates, especially in cases of immature structures that do not exhibit a distinct zonal architecture [110,111].

An additional difficulty is the significant heterogeneity of TLSs, both in terms of their structure and function. These structures can occur at various stages of development, from loose lymphocytic aggregates, through partially organized clusters, to fully mature structures containing proliferation centers. Each of these stages may have different biological and clinical significance, which further complicates their interpretation and limits the possibility of their unambiguous use as biomarkers [111,112].

Assessing the stage of a TLS remains a significant challenge. This requires the use of advanced techniques, such as IHC employing markers for B cells (CD20), T cells (CD3), dendritic cells (CD21, CD23), or markers of proliferating centers (BCL6, Ki-67). Only such an approach allows for a reliable determination of whether a given structure functions as an active center of the immune response or is merely a nonspecific inflammatory infiltrate [100,112].

Another challenge is analyzing the spatial distribution of TLS within the tumor and the TME. It has been demonstrated that the location of TLSs (e.g., intratumoral vs. peritumoral) may have significant prognostic and predictive value. However, this assessment requires the analysis of whole histological slides, which is time-consuming and difficult to standardize in daily diagnostic practice [26].

Additionally, TLS assessment is subject to significant interobserver variability, resulting from the subjective interpretation of the microscopic image and the lack of clearly defined quantitative thresholds, such as the number of structures or their density. This problem poses a significant barrier to the use of TLSs as reliable clinical biomarkers [26,109].

In response to these limitations, AI-assisted histopathology methods are becoming increasingly important. Machine learning algorithms enable the automatic identification and classification of TLS, the assessment of their spatial organization, and the quantitative analysis of immune infiltrates, which can significantly improve the reproducibility and accuracy of the diagnosis [26,110].

In summary, despite the great clinical potential of TLSs, their implementation in routine diagnostics requires the further standardization of assessment methods, the development of unambiguous classification criteria, and the integration of modern image analysis technologies.

### 6.6. AI-Based Tertiary Lymphoid Structures Detection and Quantification

Advances in AI-assisted histopathology in recent years have significantly transformed the approach to analyzing the TME, including the assessment of TLSs. Traditional histopathological analysis, based on a pathologist’s subjective evaluation of slides, is time-consuming and subject to inter-observer variability. The introduction of the WSI of histological specimens has enabled the use of advanced image analysis algorithms that significantly improve the reproducibility, objectivity, and scalability of TLS assessment [113,114].

Algorithms based on deep learning, particularly CNNs, allow for the automatic detection, segmentation, and classification of histological structures, including TLS, without the need for the manual labeling of all image elements. These models are capable of recognizing subtle morphological features and architectural patterns that may be difficult to capture in standard microscopic analysis. It has been demonstrated that the use of such methods enables not only the identification of TLS but also their quantitative assessment and classification in terms of the degree of organization and maturity [26,113,115,116].

In recent years, more advanced model architectures, such as transformer models and graph neural networks, have gained increasing importance. Transformer models enable the analysis of the global context of a histopathological image by accounting for long-range dependencies between different tissue regions. In turn, graph neural networks allow for the modeling of spatial relationships between individual cells by treating them as nodes in a graph, which enables a more accurate representation of the TME’s organization [115,117,118].

The application of these methods enables the identification of so-called immune niches, i.e., specific spatial arrangements of immune system cells and tumor cells that play a key role in regulating the immune response. In the context of TLS, the analysis of such interactions allows for a better understanding of their biological function, including their ability to initiate and sustain an antitumor response [117,118,119].

Another important area of development is the integration of imaging data with molecular (e.g., transcriptomic) and clinical data. This approach enables the construction of multidimensional predictive models that combine information on tissue morphology, cellular composition, and molecular characteristics of the tumor. These models can be used to predict patient prognosis, response to immunotherapy, and the identification of new biomarkers [113,118].

As a result, the use of AI-assisted histopathology in TLS analysis represents a significant step toward personalized medicine. The automation and standardization of the assessment of these structures can not only improve diagnostic accuracy but also enable the implementation of TLS as a reliable biomarker in everyday clinical practice.

However, AI-based TLS assessment is currently less mature than the automated analysis of more clearly defined cellular features. TLSs vary widely in size, maturation stage, cellular composition, and intratumoral or peritumoral localization, and immature TLSs may overlap morphologically with nonspecific lymphoid aggregates or Crohn’s-like lymphoid reactions [110,111,112]. This creates substantial annotation variability and makes model training dependent on the criteria used by expert pathologists. In addition, most available TLS detection models remain retrospective and require broader multicenter validation across different gastrointestinal cancer types, staining protocols, scanners, and tissue formats [113,114,115,116,117,118,119]. As a result, automated TLS quantification should be considered a promising research and translational tool, but not yet a fully standardized biomarker for routine therapeutic decision-making.

A summary of representative studies applying AI-based methods for TME assessment in GIC is presented in Table 1. The table highlights key methodological aspects, including data type, model architecture, validation strategies, and clinical relevance. Notably, while many studies report promising performance, external validation and multicenter datasets remain limited, underscoring challenges in generalizability.

## 7. Interplay Between Tumor Budding, Tumor-Infiltrating Lymphocytes, and Tertiary Lymphoid Structures

Relations between TB, TILs, and TLSs show the complex, layered organization of immune response on the local level and demonstrate that three of those TME components function as connected pathways regulating tumor progression dynamics. Therefore, TB, TILs and TLSs remain in continuous interaction instead of posing as individual histopathological features, determining both TME architecture and its invasive potential.

It has been widely reported that there is an inverse correlation between the density of TB and the TIL presence. Regions of high activity of tumor growth usually present a limited lymphocyte infiltration. It suggests that the invasion process is connected with the local silencing of immune responses. Moreover, TB actively forms TMEs by modulating pathways and interacting with stromal components, leading to limited effector lymphocyte influxes. In this context, TB may be seen as a local point of immune leakage, where the dominance of invasive mechanisms over the immune response is especially visible [126,127].

This correlation is even more meaningful in terms of stromal TIL organization. Apart from their number, the spatial distribution plays a role in their ability to countertask TB. In cases where TILs remain limited to the stroma and do not penetrate to the invasive margin, their anti-tumor functions are limited, which favors high TB. On the other hand, effective lymphocyte infiltration in the tumor’s peripheral zone may limit the budding process, indicating a bidirectional relationship in which TILs are not only inhibited by TB but may also modulate its intensity [128,129,130].

In the TME context, TLSs play the role of a structural and functional integrator of immune responses. Their presence is correlated with more effective recruitment and activation of TILs. Consequently, tumors with mature TLSs show higher density of functional TILs and lower TB, suggesting that TLSs may indirectly limit the tumor invasion process by enhancing immune response [92,131,132]. At the same time, a lack of TLSs or the presence of their immature form is linked to lower lymphocyte infiltration and a favorable environment for TB. Therefore, TLSs actively form the relations between TILs and tumor cells [112].

Interactions between TB, TLSs, and TILs should be acknowledged as a dynamic balance. On one side, there are tumors with a predominance of TB, low lymphocyte infiltration, and a lack of organized immune structures. Such environments favor tumor progression and immune evasion. On the other side, there are tumors with mature TLSs and high levels of functional TILs, where the immune response effectively limits invasive processes, leading to a reduction in TB.

In summary, TB, TILs, and TLSs form an interconnected system in which a change in one component affects the others. Analysis of these interactions allows for a better understanding of the heterogeneity of the TME and indicates that the key element in assessing tumor biology is not so much the presence of a single marker, but rather the nature of the relationships between them.

## 8. Digital Pathology and AI Pipelines for TME Assessment

The histopathological evaluation of tumor samples is based on the assessment of various prognostic factors. In daily clinical practice, one of the most laborious tasks continues to be the analysis of the TME. This is attributed to the complex relationships between tumor cells, immune infiltrates, and stroma, which sometimes are difficult to characterize in regular practice. In more complex cases, a lack of consistency between pathologists is still a problem [14].

AI-based image analysis has recently begun playing a beneficial role in TME assessment. With the increasing availability and wide accessibility of digital pathology technology, the image analysis process powered by AI has become increasingly important for diagnosis. In a typical workflow, WSI are first digitized and preprocessed, followed by model development and subsequent validation [28,30].

From a practical perspective, AI may help shorten the time required for CRC diagnoses while improving consistency between observers. In one study, AI-assisted assessment was completed in a fraction of the time compared to conventional evaluation (approximately 0.03 ± 0.01 s vs. 3 ± 5 s; *p* < 0.01), without a noticeable loss in accuracy when distinguishing between benign and malignant lesions. These findings suggest that AI can serve as a valuable adjunct in routine diagnostic workflows, particularly where both efficiency and reproducibility are important [14].

CNNs are among the most widely used models in histopathological image analysis. By capturing spatial relationships within complex tissue architectures, they perform well in tissue classification tasks and often surpass traditional machine-learning methods [14]. However, expert supervision remains essential. Broader implementation is still limited by the availability of annotated datasets, the time required for image labeling, and the lack of standardized evaluation frameworks. Errors in automated tissue classification may arise from the misclassification of lymphocytes, necrotic areas, cellular debris, or morphologically similar structures such as muscle tissue and stromal components. These errors may affect the accuracy of parameters such as the TSR, which has been associated with OS, lymph node metastasis, local recurrence, and distant metastatic spread in CRC [14].

AI has also been applied to the evaluation of TB, a recognized marker of metastatic potential and adverse prognosis in colorectal carcinoma. Tumor budding refers to single tumor cells or small clusters located within the tumor mass or at the invasive front. In routine practice, assessment involves identifying the region with the highest density of buds (the “hotspot”) and counting tumor buds within a defined microscopic field. Standardized criteria introduced by the ITBCC classify tumor budding into three categories: Bd1 (0–4 buds), Bd2 (5–9 buds), and Bd3 (≥10 buds) [24].

Assessment on H&E-stained sections can be challenging, as inflammatory infiltration or reactive stromal cells may obscure or mimic tumor buds. IHC staining with pan-cytokeratin (CK) is therefore often used to improve detection. These limitations, together with the time required for manual evaluation, support the use of computational approaches. Early image-analysis methods relied on simple features such as color and cluster size, which could lead to false-positive detections. More advanced approaches incorporate additional steps, including nuclei detection, although sensitivity to staining variability remains a limitation. Recent strategies integrate conventional image-processing techniques with deep learning models, in which candidate regions are first identified and subsequently classified using CNNs. Stain augmentation during training further improves robustness. Digital pathology also enables analysis of the spatial distribution of tumor buds across tissue sections. Density maps can illustrate areas of tumor bud accumulation, and entropy-based metrics, such as Shannon entropy, provide a quantitative measure of spatial heterogeneity within the tissue [14,24].

Deep learning models have also been applied to the automated detection of tumor buds and hotspot identification in WSI. Architectures such as Faster R-CNN and DenseNet show good agreement with expert evaluation, although automated systems may detect slightly higher numbers of tumor buds. Despite this, the results remain strongly correlated with manual assessment and may improve both interobserver and intraobserver reproducibility [27,28].

AI-based methods used in digital pathology differ in their applications, strengths, and limitations [14,24,28,29,30]. While some models are useful for the classification of histological patterns, others are better suited for tissue segmentation, object detection, tumor budding assessment, biomarker prediction, or prognostic modelling. The choice of method depends on the clinical question, available annotations, image quality and intended output. An overview of selected AI-based methods used in digital pathology and TME assessment in colorectal cancer is presented in Table 2.

Neural networks can also classify a wide range of histological tissue types, including adipose tissue, lymphocytes, mucus, smooth muscle, normal colonic mucosa, cancer-associated stroma, and tumor epithelium, with high reported accuracy in both internal and external validation settings. In addition, AI-based methods enable the estimation of the TSR, quantification of tumor-infiltrating lymphocytes, and development of novel stromal parameters associated with lymph node metastasis. However, these applications require rigorous validation before routine clinical implementation [28,29].

TB, TILs, and TLSs as biomarkers are summarized in Table 3.

## 9. Clinical Applications and Implications

The integration of AI assisted analysis into the evaluation of TMEs represents a transformative shift in the management of GICs. Clinicians are able to move beyond the constraints of traditional histopathology by leveraging computational models and achieve a more precise and reproducible diagnostic framework.

The major clinical benefit of AI assisted TME analysis is the reduction of interobserver variability. Automated deep learning models provide a level of objective quantification that manual assessment lacks. These systems have demonstrated the ability to detect tumor buds and characterize TME components with accuracy levels reaching 98% [25]. AI platforms are implemented as second opinion tools and help standardize the assessment of features like bud density and dispersion, ensuring that prognostic markers are applied consistently across different clinical settings [24,25]. Even though the TNM staging system remains the gold standard, it often fails to consider the biological heterogeneity within specific stages. AI-enhanced evaluation of the invasive front allows for superior risk stratification, especially in stage II and III CRC [49,50]. In practice, AI-derived TME metrics function as automated and standardized analogues of systems like Immunoscore and often outperform manual assessments and UICC/TNM staging in predicting survival [14,123]. Multiple nomograms and multimodal signatures now explicitly combine AI-TME scores, TNM, and clinicopathologic variables, providing higher C-indices and net reclassification improvement than TNM alone [122,134,135].

TLSs are emerging as pivotal indicators of the host’s adaptive immune response. AI platforms facilitate the precise mapping of TLS density, maturity, and spatial distribution allowing the classification of the tumors into immune-hot (high TLS/TIL presence) or immune-cold (stroma-rich) phenotypes [55,136]. In GC and CRC, the presence of mature TLSs is strongly correlated with improved DFS and serves as a surrogate for an active anti-TME [136,137]. Automated TLS quantification offers to select the patients who may benefit the most from ICIs. Beyond PD1L1 expression, the spatial arrangement of TLSs and the ratio of tumor buds to immune cell infiltrates provide a multidimensional snapshot of the TME’s readiness for immunotherapy [51,56]. AI-TME signatures, for example TMEscore, Mor-index, or Tumor Aggression-Defense Index (TADI) identify immune-inflamed profiles that complement traditional biomarkers like PDL1 or MSI/TMB [138,139]. In metastatic settings, such as HER2-amplified CRC, combined AI HER2 scores and TME density patterns have successfully separated long versus short responders to trastuzumab and pertuzumab, enabling more selective targeting [140]. In conclusion, AI assisted analysis can better identify patients with immune-hot profiles who are statistically more likely to achieve a durable response to immunotherapy, even in cases where traditional biomarkers are equivocal.

Integrated predictive models that combine TB, TILs, and TLSs into a singular “invasive front signature”, may analyze the complex interplay between invading tumor cells and the surrounding stroma and capture the molecular and morphological characteristics of tumor aggressiveness [25,47,48,55]. Consolidating that data makes it a possibility for clinicians to create personalized treatment plans that balance therapeutic intensity with the individual biological risk profile of the patient [54,120].

AI-derived metrics may support risk reclassification within conventional stages and may help identify patients who could be considered for intensified treatment in future validation-guided workflows. However, at present, these tools should be regarded as investigational or decision-support approaches rather than stand-alone determinants of adjuvant treatment. In stage II pMMR CRC, where traditional indicators are often weak, indices like the TADI and multimodal TME signatures may refine relapse risk by identifying biologically high-risk patients who should receive adjuvant chemotherapy [121,134,139,141]. These multimodal models were effective in predicting chemotherapy benefits in CRC, ensuring that the treatment is prioritized for patients with the highest risk of recurrence [134]. In GC, tools such as the DeepRisk network and radiomic-time nomograms identify which TNM II-III patients may derive the most absolute benefit from adjuvant chemotherapy after gastrectomy [124,125,142]. Furthermore, the addiction of deep-learning histology scores to ctDNA-based assessment may further improve risk stratification, but such combined approaches require prospective validation before they can guide treatment escalation in routine practice [143].

The translation of AI-TME metrics into routine clinical pipelines requires addressing practical workflow and regulatory constraints. Many current models are designed to run on routine diagnostic data, including H&E or IHC whole-slide images and standard CT scans [14,122,124,135,142]. This allows the embedding of AI analysis into existing digital pathology pipelines without requiring additional biopsies or expensive molecular testing. Automated segmentation removes manual scoring and supports standardized second-opinion services [14,123]. Despite high technical accuracy, transition to the clinic exacts prospective trials and larger, multi-center validation cohorts to ensure generalizability [14,124,144]. There is a critical need for the standardization of analytic parameters, such as the exact area size for TSR assessments [123,135].

A summary of clinical examples including the practical transition from AI-derived quantification to potential treatment decisions in different GIC stages is shown in Table 4.

## 10. Limitations and Challenges

The introduction of AI-assisted histopathological analysis has significantly expanded the possibilities for assessing the TME, but at the same time has revealed new challenges related to the identification and characterization of TLSs. A particular challenge remains their automatic detection in WSI, which is characterized by very high resolution and complex tissue architecture [26,145].

TLSs typically constitute a small percentage of the total area of a histopathological specimen, and their distribution is irregular and often limited to specific regions, such as the margin of a tumor infiltrate. Additionally, their morphology is highly variable ranging from poorly organized lymphocytic aggregates to fully developed structures containing proliferation centers which significantly hinder their unambiguous identification by both pathologists and AI models [146].

The complexity of histopathological images, the presence of numerous structures with similar morphology, and variations in the quality of slides (e.g., differences in staining or technical artifacts) further reduces the effectiveness of automated detection methods. As a result, AI models may exhibit limited sensitivity and specificity, particularly when analyzing heterogeneous data from different centers [147].

The interpretability of deep learning-based models also remains a significant limitation. Many of them function as so-called “black boxes,” meaning that the decision-making process is not fully transparent. In the context of clinical applications, this poses a significant barrier, as analysis results require the ability to be verified and justified by specialists [148,149].

Additionally, the lack of standardization in specimen preparation, scanning parameters, and analytical pipelines contributes to variability in the results obtained by different systems. These differences can lead to limited reproducibility and hinder the implementation of AI-based tools in daily diagnostic practice [30,150].

Despite these limitations, the continued development of AI-assisted histopathological analysis, combined with the growing availability of data and advances in models with increased interpretability, presents real opportunities to improve the quality and objectivity of TLS assessment in the future [26,148,149].

High TIL density generally reflects an active anti-tumor immune response and favorable prognosis, whereas histologically pronounced TB is associated with increased invasiveness and poorer clinical outcomes. Several studies have reported an inverse correlation between TB and TILs, indicating that regions with prominent TB often exhibit reduced immune infiltration. This interplay highlights the complexity of the TME and poses challenges for accurate and objective assessment, both manually and through automated AI-based histopathological analysis [151,152].

The advantages and disadvantages of manual and AI-assisted assessment in pathology have been summarized in Table 5.

## 11. Future Perspectives

The dynamic development of digital pathology and AI methods opens up new possibilities in TME analysis in GICs. Tools enabling quantitative and automated assessment of parameters such as TB, TILs, and the presence of TLSs are becoming increasingly important. Future research should aim at multi-omics integration to characterize TLS biology, international benchmarks for TLS annotation and AI detection, foundation models capturing immune spatial architecture, TLS-centered immunoprofiling for therapy selection and multimodal foundation models.

Currently, automated TB assessment is one of the most important areas of development. Digital image analysis can enable more objective and repeatable bud counting, which is particularly useful in WSI. However, the algorithms must be properly validated and standardized in order to realize the full potential of digital scoring. Thus far, most of the available studies are retrospective and involve relatively small cohorts of patients. Future studies should analyze TB in the context of TB, immune infiltration, and the molecular characteristics of the tumor, which will allow for a better understanding of the mechanisms of invasion and metastasis. In the future, TB may become a routine parameter in histopathological reports and assist in identifying patients at higher risk of lymph node metastasis and in making decisions regarding the extent of surgical or endoscopic treatment. Further standardization of assessment methods is necessary to increase the comparability of results and enable the wider application of this parameter in clinical practice [63,91,153].

Assessment of TILs density and composition may be used as a prognostic biomarker in GICs on a wider scale in the future, enabling the identification of patients who are likely to respond well to immunotherapies, particularly checkpoint inhibitors such as PD-1/PD-L1 [154]. The analysis of TILs may facilitate more personalized therapeutic strategies based on the patient’s immune profile, including adoptive T-cell therapy, and the increased efficacy of combination therapies. Furthermore, research on TILs may serve as a starting point for new therapeutic targets and the development of new ICIs that can enhance the antitumor activity of TILs and improve treatment outcomes [155]. Automatic TIL assessment allows for the detection and quantitative evaluation of TILs in histopathological images with very high accuracy, which in the future could be used for integration with digital pathology systems, support the analysis of specimens by pathologists, and provide a rapid and objective assessment of tumor immune infiltration. Despite the promising prospects, future studies require the validation of models in different centers, the use of larger and more diverse patient cohorts, and the integration of histopathological data with other biomarkers [11,154].

TLS analysis may contribute to identifying patients who will respond best to immunotherapy, predict disease progression, and design more personalized treatment strategies. The ability to induce or enhance TLS formation within the tumor may increase the effectiveness of immunotherapy and improve patient prognosis. Furthermore, TLS shows considerable variability in terms of the cellular composition and degree of maturity depending on the type of tumor and local environment, and understanding this heterogeneity is crucial for future clinical applications. However, the mechanisms underlying TLS formation in the TME are still not fully understood, so future research should focus on the genetics and immunological properties of TLS cells and the interactions between TLS and other elements of the TME including cytokines, chemokines, and stromal cells responsible for TLS induction. Technologies such as single-cell sequencing, spatial transcriptomics, and advanced TME analysis can help to better understand the role of TLS in the immune response against cancer [156,157]. It is essential to establish universal benchmarks and validation pipelines, as their current absence is one of the biggest barriers to the clinical implementation of the methods developed [158].

Advances in AI-based analysis of tissue spatial architecture may lead to an improved understanding of tissue organization, as traditional methods based solely on single-cell analysis do not fully capture the spatial organization of tissues. The integration of spatial transcriptomics and spatial proteomics with AI models will enable the identification of spatial tissue structures and interactions between cells within the TME [159].

Going forward, AI systems in pathomorphology will move towards multimodal foundation models that integrate various types of data, including WSI, radiological images, clinical, genomic, and laboratory data. Their aim will be to create a shared representation space for heterogeneous medical data, enabling the comprehensive analysis of diseases. However, the development of these models remains a challenge, as there are currently no suitable datasets that integrate all the factors mentioned [160].

One of the major limitations is the absence of a standardized and universally accepted system for assessing DR, TB, and TILs, which may cause differences in results between studies and limit the repeatability of analyses. Future studies should analyze these parameters together, which will enable the creation of integrated scoring systems, better prediction of tumor behavior, and more accurate prognostic stratification of patients [61,88].

Advances in the use of AI in pathology require the careful protection of patient privacy, as medical image data are sensitive and subject to strict regulatory frameworks. Effective protection requires a combination of technical solutions, such as federated learning and differential privacy, with appropriate ethical and legal principles, including patient consent and legal regulations [30].

Future research may enable wider use of the automated analysis of immune cell distribution in cancer tissues provided by AI models, allowing for a more objective and reproducible assessment of the TME. Another important area of research is the integration of imaging data with molecular data, which will enable a more comprehensive prognostic model for patients with GICs. Moreover, it is essential to validate AI models in large multicenter cohorts which will form the basis for the clinical application of AI tools. Further development of mentioned technologies may contribute to more precise prognostic stratification and the personalization of treatment for cancer patients [11,30,61,63,88,91,156,157].

## 12. Conclusions

The integration of AI with digital pathology represents a significant step forward in the analysis of the TME in GICs. AI tools enable a more precise, reproducible, and objective assessment. They are a great help in the assessment of key biomarkers such as TB, TILs, and TLSs, as well as their spatial relationships. This allows for better characterization of tumor biology and a more accurate evaluation of patient prognosis. What is more, usage of AI facilitates more effective patient stratification, including the identification of patients who may benefit most from immunotherapy. The integration of histopathological data with molecular and clinical information paves the way for the development of personalized medicine. However, despite its great potential, the implementation of these technologies in clinical practice requires further validation, the standardization of methods, and solutions to issues related to model interpretability and data availability. Nevertheless, AI implementation into digital pathology is crucial for further development of this medical field.

## Figures and Tables

**Table 1:** Overview of AI-based approaches for TME analysis in GIC.

Author, Year	Cancer Type	TME Component	Data Type	AI Method	Task	Validation	Cohort Size	Clinical Outcome	Key Findings
Bokhorst et al. (2023) [24]	CRC	TB	WSI	Semi-supervised deep learning-based detection	Automated detection and quantification of TB	Internal, comparison with pathologists	229 WSI	TB is linked to metastasis and poor outcome	Semi-supervised AI can detect TB with reduced annotation burden and performance comparable to experts
Bokhorst et al. (2023) [62]	CRC	TB	H&E, WSI	Fully supervised deep learning	Automated detection and quantification of TB	Internal, comparison with pathologists	229 WSI	TB is linked with worse prognosis and metastasis risk	Fully automated AI system can reliably detect TB, comparable to experts
Şeker et al. (2025) [67]	CRC	TB	H&E, WSI	Attention-based Deep Multiple Instance Learning	Automated TB classification and risk stratification	Internal, comparison with experts	hundreds WSI	TB correlates with aggressive disease and poor prognosis	Improved TB detection accuracy, reduced annotation burden
Sajjad et al. (2024) [71]	CRC	TB	H&E, WSI	Deep learning: Bayesian Multiple Instance Learning	TB detection from routine H&E-stained images	Internal	29 WSI	TB is associated with invasion, metastasis and poor survival	Proposed deep learning model improves TB detection and its reproducibility
Ben Rejeb and Yaacoubi (2025) [120]	CRC	TB	H&E, WSI	Digital pathology and semi-automated image analysis	Quantification and comparison of TB assessment	Internal	92 patients	TB correlates with aggressive tumor and worse prognosis	Used methods improve reproducibility and reduce variability in TB scoring, but still require expert supervision
Nearchou et al. (2019) [121]	CRC	TB	immunofluorescence, WSI	Automated image analysis, machine learning	Quantification of TB and spatial relationships	Internal across 3 independent cohorts	232 patients	High TB with low lymphocytic infiltration is associated with worse prognosis	Integrated spatial AI-based analysis combining TB and immune infiltration improves prognostic accuracy
Liu et al. (2023) [11]	CRC	TILs	H&E	Deep learning	Automated detection and quantification of TILs	Internal validation	Hundreds of datasets	High TILs density is linked to better survival and prognosis	Deep learning model enables more accurate and reproducible TILs quantification
Saltz et al. (2018) [26]	13 cancer types	TILs	H&E, WSI from TCGA	Deep learning (CNN-based method)	Automated mapping and spatial quantification	Internal, TCGA	5000 H&E slides	High TILs density correlates with better survival	Spatial organization of TILs correlates with prognosis
Shen et al. (2025) [83]	Locally advanced rectal cancer	TILs	H&E, molecular data	AI-based computational pathology	Prediction of response to neoadjuvant chemoradiotherapy	Clinical trial cohort	Multi cohort	AI-driven immune features can help predict treatment response	Combining immune profiling improves prediction of chemoradiotherapy response
Chen et al. (2025) [88]	GIC	TILs	H&E, WSI	Classical machine learning, computer vision	Quantification of spatial TILs distribution	Internal, cross-cohort	>1700 (2236 WSI)	Spatial TILs pattern is associated with OS	TILs spatial architecture, not just density, is stronger prognostic markers than standard TILs counts
Lim et al. (2023) [122]	Colon cancer	TILs	H&E, WSI	Deep learning (CNN-based)	Automated detection and spatial quantification of TILs	Internal, external cohorts	Training cohort: 554 patients, validation cohort: 113 patients	High spatial organization of TILs is associated with improved survival	Spatial TIL architecture improves prognostic prediction
Zhao et al. (2022) [95]	Colon cancer	TLS	H&E, WSI	AI-based image analysis	Automated quantification of CLR density	internal, comparison with experts	Training cohort: 279, validation cohort: 194	High CLR density associated with improved survival and favorable prognosis	AI-quantified CLR density is a prognostic biomarker, superior to manual assessment
Le Rochais et al. (2025) [116]	CRC	TLS	H&E, WSI	Deep learning	Detection and classification of TLS	Internal	656 patients	Higher density of TLS is associated with improved prognosis	AI-based TLS detection enables reproducible and objective quantification of TLS
Carvalho et al. (2025) [123]	CRC	TSR	H&E, WSI	AI-based image analysis	Automated quantification of TSR and prognostic stratification	Internal, survival analysis	1317 patients	Low TSR is associated with worse prognosis	AI-based TSR quantification provide objective, reproducible prognostic biomarker
Chen et al. (2022) [124]	Gastric cancer	TME	H&E	Machine learning-based computational pathology	Quantification of TME and prediction of survival outcomes after gastrectomy	Internal	415 patients	AI-derived TME score is associated with OS and prognosis	Integrated AI-based TME scoring system improves prognostic stratification compared to conventional clinicopathological factors
Tian et al. (2024) [125]	Gastric cancer	TME	H&E, WSI	Deep learning	Prediction of prognosis and response to neoadjuvant chemotherapy	Internal, external TCGA	multi-cohort	Deep learning methods help predict treatment response	Deep learning methods provide strong prognostic performance

**Table 2:** Overview of AI-based methods used in digital pathology and TME assessment in colorectal cancer.

Model	Best Suited for	Main Application	Advantages	Limitations	Ref.
Convolutional neural networks (CNNs)	When the task requires recognition of histological image patterns	Classification of tissue regions, tumor/stroma recognition, TME-related feature extraction	Strong performance in image-based pattern recognition, can learn morphological features directly from WSI patches	Require large annotated datasets, may overfit on small cohorts, sensitive to staining variation, scanner differences and artifacts and limited interpretability	[14,28,30]
Segmentation-based deep learning models	When precise localization of tissue compartments is needed	Delineation of tumor, stroma, immune-rich regions, necrosis or other regions of interest	Provide spatial information and enable compartment-based quantification	Require detailed pixel- or region-level annotations, annotation is time-consuming and observer-dependent, performance may decrease with poor staining	[14,30]
Object detection-based models	When individual small structures need to be counted or localized	Detection of tumor buds, immune cells or other cellular objects	Useful for quantitative and spatial analysis, may reduce manual workload	Small targets are difficult to detect, false-positive detections may occur and results depend strongly on image quality	[14,24,30]
Semi-supervised teacher–student models	When expert-labelled data are limited but unlabeled WSI data are available	Automated tumor bud detection in cytokeratin-stained WSI	Reduces the need for exhaustive expert annotations, can use pseudo-labels to enlarge training data	Pseudo-labels may be less accurate than expert annotations, errors may propagate during training and weak or variable staining and necrotic areas may reduce accuracy	[24]
Weakly supervised/slide-level prediction models	When only slide-level or patient-level labels are available	Prediction of prognosis, molecular status, treatment response or AI-derived biomarkers	Reduces the need for detailed manual annotation, useful for large retrospective cohorts	May learn dataset-specific or non-biological correlations, limited localization of relevant histological features and external validation is essential	[28,29,30]
AI-based MSI or mutation pre-screening models	When rapid triage for confirmatory molecular testing is needed	Prediction or pre-screening of MSI status or selected gene alterations from H&E slides	May support case prioritization and precision oncology workflow	Should not replace standard molecular testing without validation, some single-gene predictions, such as BRAF/RAS, may remain inferior to sequencing; commercial tools require independent validation	[29]
Multiscale/multimodal AI models	When the clinical question depends on information from several tissue scales or data sources	Integration of cellular, tissue-level, WSI, clinical and/or molecular data	May better capture the biological complexity of CRC and TME	Computationally demanding, requires harmonized multimodal datasets and difficult to interpret and validate	[30]
Explainability methods/interpretable AI	When AI output may influence clinical decisions and needs to be reviewed by pathologists	Heatmaps, attention maps, feature attribution, uncertainty or confidence assessment	May improve transparency and clinical trust	Explanations may be incomplete or misleading, highlighted regions do not always prove biological causality and requires clinical evaluation	[28,30]

**Table 3:** TB, TILs, and TLSs as biomarkers in GIC.

Biomarker	Definition	Spatial Context	Clinical Significance	AI Applications	Ref.
Tumor budding	Single cells/cluster up to 4 cells at invasive front of tumor center	Mainly invasive front	Worse prognosis, invasion, metastasis	Bud detection, grading	[24,25,47,48,49,50,51,52,53,54,55,56,57,58,59,60,61,62,63,64]
Tumor-Infiltrating Lymphocytes	Lymphocytes within tumor and stroma	Tumor center, invasive margin, stromal compartments	Often better prognosis, immunotherapy relevance	Density estimation, spatial mapping	[26,27,28,29,30,73,74,75,76,77,78,79,80,81,82,83,84,85,86,87,88,89,133]
Tertiary Lymphoid Structures	Organized ectopic lymphoid aggregates	Intratumoral/peritumoral/invasive margin	Usually favorable prognosis	Detection, maturity classification	[90,91,92,93,94,95,96,97,98,99,100,101,102,103,104,105,106,107,108,109,110]

**Table 4:** Examples of AI-TME impact on clinical decisions.

Clinical Context	AI-TME Metric Utility	Potential Decision Change	Ref.
Stage II CRC	TADI/Multimodal TME signature	Reclassify to high-risk, recommend adjuvant chemotherapy	[121,134,139,141]
Stage II-III GC	Radiomic/Immunoscore/Nomogram	Identify absolute benefit from adjuvant chemotherapy	[124,125,142]
MRD-negative CRC	Deep-learning histology score	Offer chemotherapy despite negative ctDNA	[143]
mCRC (HER2+)	AI HER2 QCS + TME density	Prioritize targeted therapy in TME-low profiles	[140]

**Table 5:** Manual and AI-assisted assessment in pathology: advantages and disadvantages.

Type of Assessment	Advantages	Disadvantages	Ref.
Manual	- adjusting to challenging cases - considering clinical context - assessment of wider histopathological picture - high experience - gold standard	- intraobserver variability - lower risk stratification - time-consuming - high heterogeneity of the structures - lack of consistency	[14,16,147,148,149,151,152]
AI-assisted	- more precise diagnostic - reproducible framework - objective quantification - higher accuracy - help with creating personalized treatment plans	- challenges with identification of poorly developed structures - problems with low quality of histopathological images - lack of standardization - detects what has been taught	[14,16,25,49,50,146,147,148,149,151,152]

## Data Availability

No new data were created or analyzed in this study. Data sharing is not applicable to this article.

## Abbreviations

The following abbreviations are used in this manuscript:

AI	Artificial Intelligence
CAF(s)	Cancer-Associated Fibroblasts
CLR	Crohn’s-like lymphoid reaction
CNN(s)	Convolutional Neural Network(s)
CRC	Colorectal Cancer
CTL(s)	Cytotoxic T Lymphocytes
DFS	Disease-Free Survival
DR	Desmoplastic Reaction
ECM	Extracellular Matrix
EMT	Epithelial–Mesenchymal Transition
GIC	Gastrointestinal Cancer
HEV(s)	High Endothelial Venules
H&E	Hematoxylin and Eosin
ICB	Immune Checkpoint Blockade
ICI(s)	Immune Checkpoint Inhibitors
IHC	Immunohistochemistry
IL	Interleukin
IM	Invasive Margin
ITBCC	International Tumor Budding Consensus Conference
MSI	Microsatellite Instability
MSI-H	Microsatellite Instability-High
MSS	Mutations and microsatellite stability
NK cells	Natural Killer cells
OS	Overall Survival
PD-1	Programmed Cell Death Protein 1
PD-L1	Programmed Death-Ligand 1
TADI	Tumor Aggression-Defense Index
TAM(s)	Tumor-Associated Macrophages
TAN(s)	Tumor-Associated Neutrophils
TB	Tumor Budding
TCGA	The Cancer Genome Atlas
TIL(s)	Tumor-Infiltrating Lymphocytes
TLS	Tertiary Lymphoid Structures
TME	Tumor Microenvironment
TNM	Tumor, Node, Metastasis staging system
TSR	Tumor–Stroma Ratio
VEGF	Vascular Endothelial Growth Factor
WSI	Whole-Slide Imaging
